# American Medical Association

**Published:** 1848-04

**Authors:** 


### AMERICAN MEDICAL ASSOCIATION.

It is, we suppose, generally known to our readers, that the *National Medical Convention*, at the close of its second meeting, in Philadelphia, May 1847, resolved itself into a permanent society, under the title of AMERICAN MEDICAL ASSOCIATION; and adjourned to meet in Baltimore on the first Tuesday in May of the present year. It is to remind the profession in the West, of the meeting which will take place within a few weeks of this time, that we write this notice. As the proceedings of the Convention may not have reached many parts of the country, we extract those portions of the second section of the Constitution, which relate to the election of members.

"The members of this institution shall collectively represent and have cognisance of the common interests of the medical profession in every part of the United States, and shall hold their appointment to membership either as delegates from local institutions, as members by invitation, or as permanent members.

"The delegates shall receive the appointment from permanently organized medical societies, medical colleges, hospitals, lunatic asylums, and other permanently organized medical institutions of good standing in the United States. Each delegate shall hold his appointment for one year, and until another is appointed to succeed him, and shall participate in all the business and affairs of the association.

"Each local society shall have the privilege of sending to the association one delegate for every ten of its regular resident members, and one for every additional fraction of more than half of this number. The faculty of every regularly constituted medical college or chartered school of medicine shall have the privilege of sending two delegates. The professional staff of every chartered or municipal hospital containing a hundred inmates or more, shall have the privilege of sending two delegates; and every other permanently organized medical institution of good standing shall have the privilege of sending one delegate.

"The members by invitation shall consist of practitioners of reputable standing, from sections of the United States not otherwise represented at the meeting. They shall receive their appointment by invitation of the meeting, after an introduction from any of the members present, or from any of the absent permanent members. They shall hold their connection with the association until the close of the annual session at which they are received, and shall be entitled to participate in all its affairs as in the case of delegates. The permanent members shall consist of all those who have served in the capacity of delegates, and of such other members as may receive the appointment by unanimous vote."

We ardently hope, that the various schools and societies of the interior, will send delegates, not only to the first, but every subsequent meeting of the society; and that many unofficial members of the profession will attend and become *members by invitation!* A number of Reports on important subjects will be read and discussed, and provision is, also, made for the reception of papers on medical and scientific subjects, from all or any member of the Association. Thus the meeting cannot fail to be one of deep and profitable interest, not only to its members, but the profession at large.

